# Floodplain Forests Are Sensitive to Salt-Intrusion During Summer Droughts When Dominated by *Salix*

**DOI:** 10.1007/s12237-026-01726-1

**Published:** 2026-05-07

**Authors:** Eleonora Saccon, Suzanne J. M. H. Hulscher, Tjeerd J. Bouma, Johan van de Koppel

**Affiliations:** 1 Department of Estuarine and Delta Systems, Royal Netherlands Institute for Sea Research (NIOZ), Korringaweg 7, Yerseke, 4401 NT Netherlands; 2 Department of Water Engineering and Management, Faculty of Engineering Technology, University of Twente, Enschede, Netherlands; 3 Faculty of Geosciences, Department of Physical Geography, Utrecht University, Utrecht, 3508 TC The Netherlands; 4 Conservation Ecology Group, Groningen Institute for Evolutionary Life Sciences, University of Groningen, Groningen, 9700 CC Netherlands

**Keywords:** Alluvial forests, Climate change, Estuaries, *Salix alba*, *Alnus glutinosa*, Storm surge

## Abstract

Climate change and human activities are increasing salt intrusion into estuaries worldwide. This is threatening the last remaining temperate freshwater floodplain forests, which are vulnerable to sea level rise and more extreme river discharges. In temperate regions, salt intrusion events typically occur during summer droughts and/or winter storm surges. While such shift in timing of salt intrusion events could influence vegetation differently based on their phenological cycle, the seasonal impact on freshwater vegetation remains poorly understood. In this study, we exposed two dominant freshwater floodplain-forest tree species, *Salix alba* and *Alnus glutinosa*, to saltwater stress for one week during each temperate season: spring, summer, autumn, and winter. Our results show that *Salix alba* is highly sensitive to salt stress in spring and summer, growing up to 96% less in hight and up to 95% less in diameter, and reduced recovery the next spring (Leaf Health Index of -1 compared to 1 in the control treatment). On the other hand, exposure in autumn and winter had little effect. In contrast, *Alnus glutinosa* showed consistent resistance and recovery across all seasons. These findings highlight that assessments of flood-plain forest vulnerability must consider not only species-specific salt tolerance, but also the timing of salt intrusion, as events during the spring or summer growing season pose a particular risk.

**Supplementary Information:**

The online version contains supplementary material available at 10.1007/s12237-026-01726-1.

## Introduction

Estuaries and coastal areas around the world are increasingly impacted by salt intrusion events, driven by climate change and human activities (Costa et al., 2023; Hanley, Bouma et al., 2020a; Herbert et al., 2015; Lee et al., 2024). Climate change contributes to this issue through rising sea levels, more frequent storm surges in winter, and prolonged extreme droughts (low river discharge periods) in summer. The two processes have different effects: the first two encourage seawater to move upstream, while the latter reduces the freshwater flow from rivers (Intergovernmental Panel on Climate Change, 2023; Jones et al., 2024; Lee et al., 2024). Additionally, human activities such as dredging of navigation channels, groundwater withdrawal, and river diversion can further intensify salt intrusion events (Herbert et al., 2015; Tully et al., 2019). Increasing occurrence of salt intrusion events will threaten the survival of freshwater wetlands, including the already rare and globally endangered tidal floodplain forests (Havrdova et al., 2023). Once widespread across many temperate-zone estuaries, these ecosystems are now restricted to small, fragmented areas (Havrdova et al., 2023; Verhoeven, 2014). Despite their decline, floodplain forests are increasingly recognized for the ecosystem services they provide, such as flood protection and carbon sequestration (Riis et al., 2020; Schuster et al., 2024; van Wesenbeeck et al., 2022), and are being implemented as nature-based solution (Borsje et al., 2011; Jakubínský et al., 2021). Acknowledging the vital role these ecosystems play in supporting biodiversity and providing ecosystem services, floodplain forests are increasingly restored and protected. For example, the European Union has implemented protections for the remaining European floodplain forests under the European Habitats Directive, aimed at their conservation and restoration (Council Directive 92/43/EEC, 1992; Verhoeven, 2014).

Many species currently employed in nature-based solutions were originally selected based on historical environmental conditions and past management objectives. Yet, climate change and direct human activities are exposing ecosystems to unprecedented and simultaneous stressors (Intergovernmental Panel on Climate Change, 2023; Lemm et al., 2021; Palmer et al., 2009), which could render nature-based solutions unsuitable for these new conditions. Existing management policies that prioritize maintaining the current, often single-species, forest composition may inadvertently increase vulnerability to salt intrusion compared to more diverse forests. Given the considerable variation in salt stress sensitivity both within and between species (Conner et al., 1997; Hanley, Bouma et al., 2020a; Saccon et al., 2025; Tolliver et al., 1997), a more adaptive approach that promotes species diversity could enhance ecosystem resilience. Encouraging natural succession and increasing biodiversity have been proposed as effective strategies (Messier et al., 2019) to facilitate a gradual transition from single-species freshwater forests to mixed forests that include both freshwater and brackish-tolerant species. For example, some researchers advocate for the introduction of more salt-resistant species to guarantee their long-term functionality as nature-based solution (Van Coppenolle & Temmerman, 2019). Such an approach could mitigate large-scale tree mortality and strengthen ecosystem resilience against multiple climate change-induced stressors (Isbell et al., 2015; Messier et al., 2019; Mori et al., 2013).

Building more resilient floodplain forests starts with improving our understanding of how sensitive they are to stresses such as salt intrusion. Salt stress can be either acute, characterized by a short and intense pulse that is subsequently alleviated, or prolonged, in which salts are not removed from the soil following the intrusion event (Krauss et al., 2007). Beyond stress duration, the timing of salt intrusion events is also critical. In temperate areas, salt intrusion mostly occurs either during summer droughts or winter storm surges (Bolotin et al., 2022; Lee et al., 2024; Wegman et al., 2024). Deciduous trees grow actively during spring and summer, but drop their leaves and enter a state of dormancy in autumn and winter (Vitasse et al., 2014). Therefore, it may be expected that salt intrusion during a summer drought could have a profoundly negative impact on plants, whereas a winter storm surge might have little to no effect. While the timing of salt intrusion —whether during a summer drought or a winter storm surge— may greatly affect how plants respond to salt stress, to our knowledge only few studies have addressed this topic. One study on *S. alba* found that the combination of winter flooding and salt stress during winter had no effect on cuttings growth in the following spring (Markus-Michalczyk et al., 2024). Outside of salt intrusion studies, studies on other stresses like droughts, frost or higher precipitation events all highlighted how the timing of environmental stress lead to differential shifts in phenology, biomass and growing habit, with stronger effects in summer than in winter (Charrier et al., 2021; Zedler et al., 1986; Zeppel et al., 2014). The seasonal timing of salt intrusion likely plays a considerable role on species-specific growth responses and survival chance. Gaining a clearer understanding of when salt intrusion poses a threat to which tree-species is thus essential for protecting the last remaining floodplain forests and ensuring their continued functionality as effective nature-based solutions.

In this paper we tested the effect of seasonality of salt stress on the growth and recovery of two common freshwater trees of European floodplain forests: white willow *S. alba* and black alder *A. glutinosa*. For each temperate season (spring, summer, autumn, or winter), a different set of trees were subjected to salt stress at two different salinities (5 or 20 PSU) for one week. When not subjected to salt exposure, all trees were kept in freshwater until the following spring when we measured tree recovery. We hypothesised that: (1) high salt stress in spring and summer prevents plants from recovering, whereas salt stress in autumn and winter has only minor long-term effects due to winter dormancy; (2) the alder species *A. glutinosa*, which was formerly dominant in European freshwater flood forests, might have been better adapted to salt intrusion than the willow species *S. alba*, which is cultivated for commercial purposes and is currently dominant.

## Methods

### Study Area

This study focusses on the tidal freshwater floodplain forests of the densely populated Rhine-Meuse estuary in north-west Europe. Here the original multi-species floodplain forests have largely been replaced since the 14th century by white willow (*Salix alba*) plantations, whose branches were used in basketry and land reclamation (Paalvast & van der Velde, 2014; Struyf et al., 2009). These monocultures have supplanted the historically dominant black alder (*Alnus glutinosa*), coexisting willow species (*Salix alba* and *Salix fragilis*), and reed beds (*Phragmites australis*) that once characterized these landscapes (Barendregt et al., 2006; Pierik et al., 2022; Vos, 2015). These forests were created by propagating willow cuttings, a practice that has been used for centuries and is still in use today. This results in forests that are essentially made up of clones of a small number of individuals. After WWII, the economic value of white willow plantations has declined, and today only small portions of these areas are maintained as managed artificial wetlands, serving limited roles in conservation (Bijlsma et al., 2009; Paalvast & van der Velde, 2014; van den Bergh et al., 2009). Many sections are left unmanaged and show no evidence of regeneration through seedling recruitment. Instead, only the vegetative propagation of existing plants persists (Bijlsma et al., 2009). This suggests that both managed and unmanaged stands may all originate from just a few individuals and have thus low genetic diversity. Nevertheless, white willows have found a new role in nature-based solutions. An example is their use in the Noordwaard floodplain dike protection project in the Netherlands, where they contribute to enhancing flood defence (Borsje et al., 2011). The reference values used in this experiment were based on salinity data collected by Saccon et al. (2025) in a freshwater tidal forest in the Dutch Rhine–Meuse estuary. Measured salinity ranged from 0 to 11 PSU, with the upper extreme representing roughly one‑third the salinity of open seawater (i.e. 30 PSU). During the monitoring period, salinity exceeded 5 PSU on only 5% of the days, indicating that the site is predominantly governed by freshwater conditions. Nevertheless, episodic salt‑water intrusions raise salinity to around 5 PSU— matching the concentration used in the present experiment. Salt intrusion in the Rhine–Meuse estuary has been documented as far as 44.2 km upstream (Wegman et al., 2024). Because of this, the freshwater forest stand examined by (Saccon et al., 2025), located 23 km upstream from the river mouth, is regularly exposed to these intrusion events.

### Experiment Design

From April 2022 to May 2023, we conducted a mesocosm experiment to test the effects of seasonality of salt intrusion on juvenile (around 2–3 years old) trees of *Salix alba* L. and *Alnus glutinosa* (L.) Gaertn. We opted for juvenile trees over adult trees to obtain a conservative estimate of the species’ salt tolerance, as earlier research indicated that salt tolerance improves with tree age, with juvenile trees typically being more sensitive than mature ones (Kozlowski, 1997; Niknam & McComb, 2000). The trees were bought at a local tree nursery (Boomkwekerij De Bruyn bv, Begijnendijk, Belgium) and brought to the Royal Netherlands Institute for Sea Research (NIOZ) in Yerseke, the Netherlands. Because the experiment required a large number of similar individuals, collecting material directly from protected natural forests was not permitted. Moreover, propagating *Alnus* *glutinosa* from cuttings is considerably more difficult than for *Salix alba* (Novotná & Štochlová, 2013), making reliable rooting of *A. glutinosa* under our experimental conditions impractical. Consequently, we sourced the trees from a local nursery that supplies domestically produced stock. The supplier confirms that all plants are cultivated within the country and originate either from the immediate vicinity of the nursery or from the area around Zundert, the Netherlands. Both areas are freshwater, which suggests that the individuals were not previously subjected to salt stress. Please note that trees sourced from a local nursery may exhibit low genetic variability. Similar to standardised models in genomic research, this uniformity ensures a high degree of morphological consistency, guaranteeing that any observed variations are due to experimental variables rather than underlying genetic differences.

We potted one tree per pot in 20 × 20 × 24 cm pots using planting soil with 10% organic carbon (Terrafin BV, Grijpskerke, Netherlands). The pots were made of low-density polyethylene, which has limited chemical reaction to water chemistry variations. Water entered the soil through drainage holes at the bottom of the pots. The experiment consisted of nine treatment combinations: a freshwater control and two salinity levels (5 PSU and 20 PSU) that were used to give a salinity pulse in each of the four temperate seasons (spring 2022, summer 2022, autumn 2022 and winter 2023). For every treatment (i.e., control, 20PSU-spring, 20PSU -summer, 20PSU -autumn, 20PSU -winter, 5PSU-spring, 5PSU -summer, 5PSU -autumn, 5PSU -winter), we used four individuals of *Salix alba* and four individuals of *Alnus glutinosa*, giving eight trees per treatment. Each tree was exposed to its assigned salinity condition for one week. The 5 PSU salinity was selected because it was previously indicated as a survival threshold for freshwater trees (Saccon et al., 2025). The 20 PSU concentration was chosen to be high enough to damage freshwater trees even after one week of exposure, simulating the effect of acute stress such as a drought or storm surge event (Krauss et al., 2007). The seasonal treatments were conducted until February 2023, afterwards all plants were kept in freshwater until May 2023 to measure recovery during the following growing phase. We randomly placed four trees of each species in mesocosms, resulting in 8 trees per mesocosm. The mesocosms were placed outdoors and exposed to natural environmental conditions. Treatments used filtered seawater collected from the nearby Eastern Scheldt estuary, which is a sea-arm with an open exchange with the North Sea. We blended the Eastern Scheldt water with tap water to obtain the right salinity. As a result, this setup better reproduced the natural salt-stress conditions experienced by plants in the study area which originates from the North Sea, which we regard preferable compared to using other products to create salt-treatments (Hanley et al., 2020b). The pots were continuously submerged at a fixed water level (i.e., 12 cm high) up to half the height of the pot (i.e., 24 cm high), both during the salinity exposure and outside of the salinity exposure. Salt concentrations were checked with a salinity meter (HI98319 Hanna Marine Salinity Tester, Nieuwegein, Netherlands). Saltwater was added to the mesocosms during the first week of the month, and at the end of the week, the saltwater was pumped out, the mesocosms were rinsed until the water became fresh, and then they were refilled with freshwater.

### Quantifying Plant Responses

We took monthly measurements of plants morphological properties: tree height, diameter, and number of leaves, similarly to the methods described in (Saccon et al., 2025). Tree height and diameter were measured at the rim of the pot, to consider variations in soil levels between pots and to have comparable measures between individuals. To account for potential variability in leaf numbers between branches, we counted the leaves in two 10 cm sections along the trunk, each encompassing several branches. The first section was located 10 to 20 cm from the highest point of the plant, and the second was 10 to 20 cm above the edge of the pot. We categorized the leaves in three categories to estimate the health of the plants: completely healthy if without any visible damage, damaged if any signs of damage or colour alteration were present, and dead if no green parts were present. Additionally, we measured both soil and water salinity monthly throughout the whole experiment, using an EC meter (EC1200, Nieuwkoop BV, Aalsmee, Netherlands), which is suited for measuring directly in water and in water saturated soils like our inundated pots. Soil salinity was measured both in the first few centimetres of top-soil and at the bottom of the pot to consider the effect of submergence on soil salinity.

At the end of the experiment in May 2023, we harvested six plants per treatment to retrieve the above- and belowground biomass. Since measuring biomass involved sacrificing the trees, it was only possible to measure it at the end of the experiment and compare the results of the treatments with the control. The belowground biomass was divided in two sections at half pot height (i.e., upper and lower 12 cm) to consider the influence of submergence on root growth. We washed and sieved the belowground biomass to retrieve the roots. Subsequently, both above- and belowground biomass were weighed, dried at 60 °C to constant mass and weighed again to get fresh and dry weight.

### Calculating the Leaf Health Index and Statistical Analysis

To understand the effect of seasonal salt intrusion on plant growth, we calculated the difference in diameter and height between the first measure (April 2022) and the last measure (May 2023). For the leaf measures, we adopted the index presented in Saccon et al. (2025) to allow comparisons between species with different growing habits and number of leaves. This so-called Leaf Health Index, weights the number of counted leaves based on their health: 1 for completely healthy leaves (as their entire surface can photosynthesize), 0.5 for damaged (as they contribute less to plant health than healthy leaves), and -1 for dead leaves (indicating stress; thus, a decrease in the index value reflects the impact of environmental stress on plants). The weighted leaves are then summed and the total divided by the total number of leaves, ensuring the index ranged from 1 (all healthy leaves) to -1 (no leaves present or all dead) (Eq. 1). If no leaves were present, the plant was assigned an index value of -1. All weights were nonzero to ensure all counted leaves were included in the calculation. By comparing the index values of the salinity treatments with those of freshwater conditions, we assessed the effect of salt stress on the plants and exclude damage from other sources, such as insects. If increased stress from salinity treatments led to more insect damage, this was considered part of the indirect effects of salt stress and included in the index calculation.

1 $$\begin{array}{c}\text{Leaf Health Index}=\\ \left\{\begin{array}{c}\frac{\text{1 * healthy leaves + 0.5 * damaged leaves - 1 * dead leaves}}{\text{ total number of leaves}},\\ \begin{array}{c} |\text{ total number of leaves}>0\\ \begin{array}{c}-1,\\ | \text{total number of leaves}=0\end{array}\end{array}\end{array}\right.\end{array}$$

All statistical analyses were performed with R Statistical Software (v4.2.2; R Core Team, 2022). To facilitate comparisons between water salinity and soil salinity we converted soil salinity from EC to PSU using the wql package in R (Jassby et al., 2017). This package implements algorithms developed by Fofonoff and Millard (1983) and Poisson and Gadhoumi (1993). The biomass values (aboveground biomass and belowground biomass in both soil layers) were first log-transformed to achieve normality and homoscedasticity. To assess whether baseline traits differed among individuals prior to the salinity treatment, we fitted separate two‑way ANOVAs for Leaf Health Index, tree diameter, and tree height, with species, treatment, and their interaction included as fixed factors. Differences in baseline traits were primarily associated with species rather than treatment (Table S1). *Salix alba* exhibited larger mean tree diameter than *A. glutinosa*,(10.97 mm vs. 9.41 mm, F_1,48_ =4.305, *p* = 0.043, *n* = 64). Mean tree height differed substantially between species, with *S. alba* averaging 116.94 cm compared to 74.97 cm for *A. glutinosa* (F_1,48_ =257.211, *p* < 0.0001, *n* = 64). Leaf Health Index slightly differed between species, averaging 0.78 in *S. alba* and 0.53 in *A. glutinosa* (F_1,32_ =12.335, *p* = 0.001, *n* = 48). These metrics showed no detectable differences between treatments within each species, consistent with a random allocation of individuals to mesocosms prior to salinity exposure.

To understand the effect of the treatments, we performed a two-way ANOVA on all the parameters (tree growth in diameter and height, the measures of Leaf Health Index and soil salinity in both layers taken at the end of the experiment, log-transformed biomass values) to compare differences between treatments, with species, seasonality, and their interactions as response variables. An additional ANOVA was performed on the same parameters for each single species, with seasonality as response variables. Additionally, we applied a post-hoc Dunnet test (Dunnett, 1955) on all parameters for each single species, to see if statistically significant differences were present between the salinity treatments and the control.

## Results

### Observed Salinity Levels in the Seasonality Treatments

**Fig. 1:**
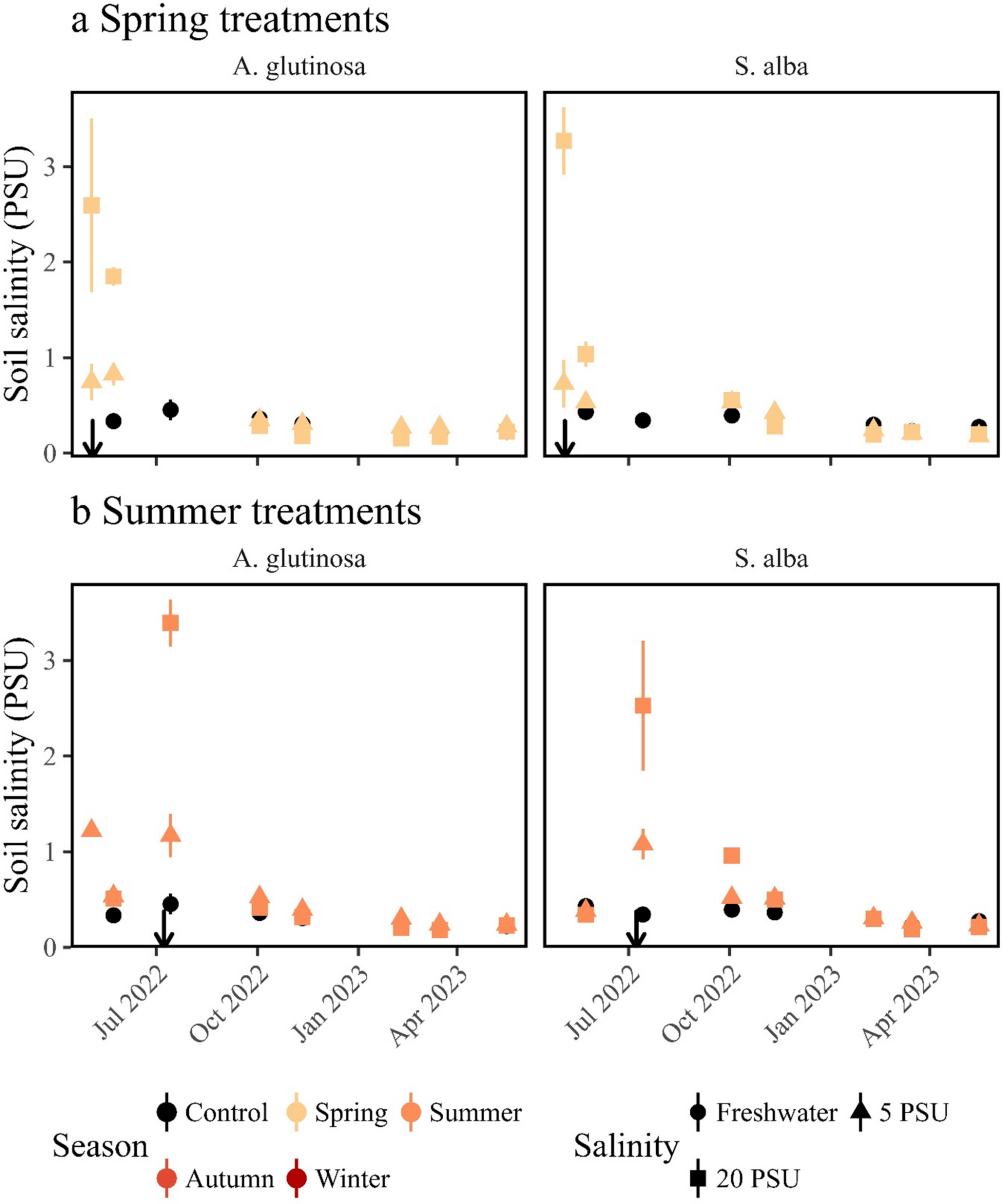
Soil salinity (practical salinity units, PSU) measured in the lower half of the pots for the two tree species. Error bars represent mean ± standard error. (**a**) Spring treatment: soil‑salinity values for the control (black circles) and the two salinity levels (triangles = 5 PSU; squares = 20 PSU). The left panel shows Alnus glutinosa; the right panel shows *Salix alba*. The arrow indicates the date the spring salinity pulse was applied (04 May 2022). (**b**) Summer treatment: same format as panel a, displaying the soil‑salinity response after the summer salinity pulse (08 July 2022). Left panel: *A. glutinosa*; right panel: *S. alba*

**Fig. 2:**
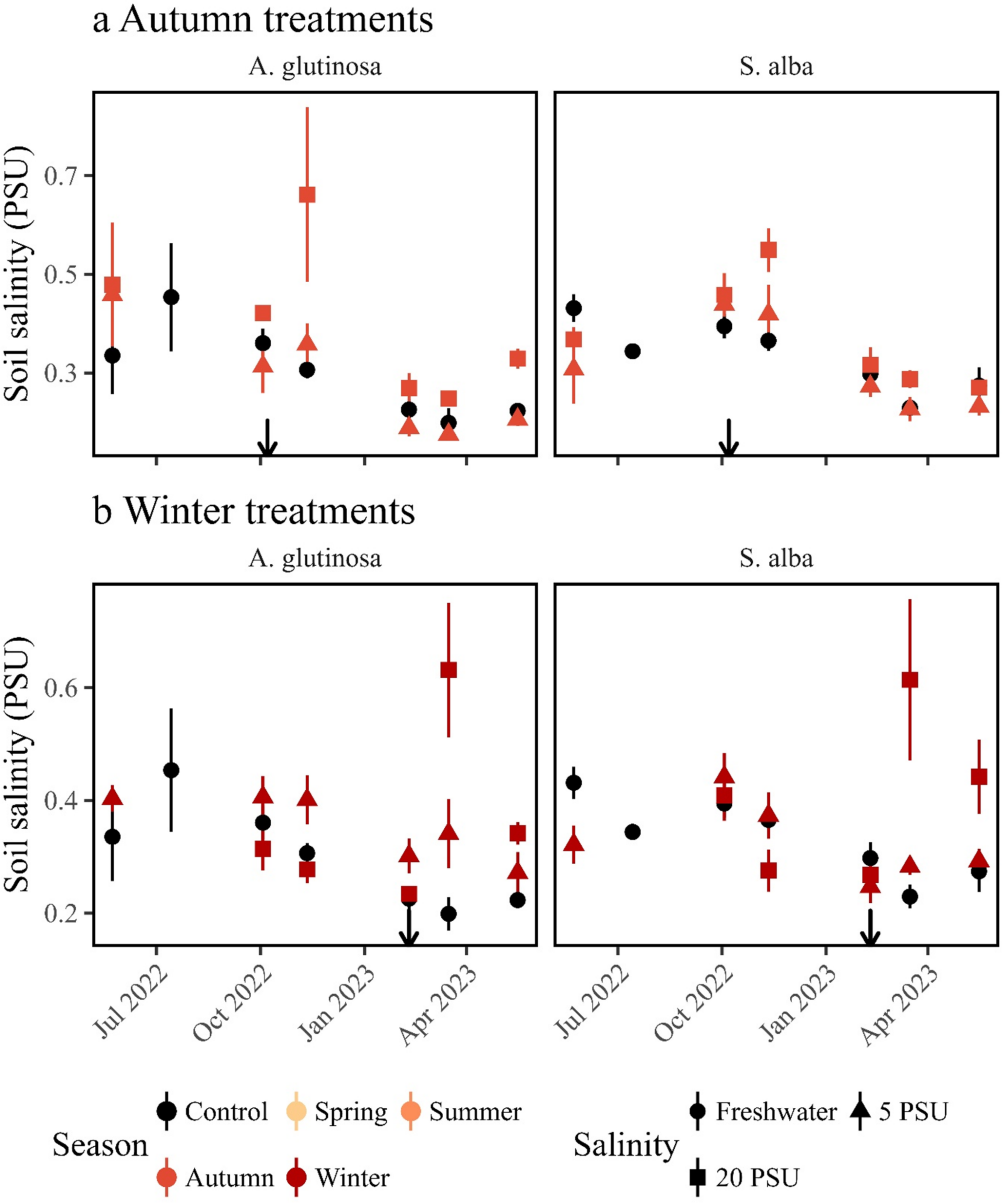
Soil salinity (PSU) measured in the lower half of the pots for the two tree species. Error bars represent mean ± standard error. (**a**) Autumn treatment: soil‑salinity values for the control (black circles) and the two salinity levels (triangles = 5 PSU; squares = 20 PSU). The left panel shows *Alnus glutinosa*; the right panel shows *Salix alba*. The arrow indicates the date the spring salinity pulse was applied (04 May 2022). (**b**) Winter treatment: same format as panel a, displaying the soil‑salinity response after the summer salinity pulse (08 July 2022). Left panel: *A. glutinosa*; right panel: *S. alba*

An increase in salinity was observed during the salt exposure in the spring and summer 2022 treatment, followed by a return to freshwater levels after treatment completion. Although salinity was not directly measured during the salinity exposure in the autumn 2022 and winter 2023 treatments, pre- and post-treatment values suggest a similar pattern (Figs. 1 and 2). At the end of the experiment on May 16, 2023, all treatments exhibited soil salinity within the freshwater range, specifically between 0 and 1 mS (Figure S1A, Figure S1B). Even the seemingly higher values in the 20 PSU treatment, fell within this range (soil salinity upper layer: F_8,54_=12.23, *p* < 0.0001, *n* = 72, Table 1), showing that all treatments returned to freshwater levels at the end of the experiment.

**Table 1:** Two-way ANOVA results on the last measures of the soil salinity in both soil layers, the last measures of Leaf Health Index, the tree growth in diameter and height (difference between the last and the first measure), the aboveground biomass, the upper and lower half of the belowground biomass

Measure	Term	DF	SS	MS	F	*P*	*N*. obs
Leaf Health Index	treatment	8	12.752	1.594	25.473	< 0.0001	60
	species	1	1.741	1.741	27.821	< 0.0001	60
	treatment: species	8	6.805	0.851	13.592	< 0.0001	60
Soil salinity (mS), lower 12 cm	treatment	8	0.841	0.105	8.324	< 0.0001	72
	species	1	0	0	0.011	0.917	72
	treatment: species	8	0.229	0.029	2.269	0.036	72
Soil salinity (mS), upper 12 cm	treatment	8	1.005	0.126	12.228	< 0.0001	72
	species	1	0.071	0.071	6.906	0.011	72
	treatment: species	8	0.11	0.014	1.334	0.247	72
Tree diameter	treatment	8	405.188	50.649	7.995	< 0.0001	72
	species	1	0.994	0.994	0.157	0.694	72
	treatment: species	8	25.179	3.147	0.497	0.853	72
Tree height	treatment	8	2586.694	323.337	5.403	< 0.0001	72
	species	1	747.556	747.556	12.492	0.001	72
	treatment: species	8	2068.194	258.524	4.32	0	72
Dry aboveground biomass (g)	treatment	8	16.275	2.034	15.641	< 0.0001	54
	species	1	4.379	4.379	33.666	< 0.0001	54
	treatment: species	8	5.554	0.694	5.338	0	54
Dry belowground biomass (g), lower 12 cm	treatment	8	10.445	1.306	5.054	0	50
	species	1	1.317	1.317	5.098	0.031	50
	treatment: species	7	6.519	0.931	3.605	0.005	50
Dry belowground biomass (g), upper 12 cm	treatment	8	46.698	5.837	12.627	< 0.0001	54
	species	1	9.362	9.362	20.251	< 0.0001	54
	treatment: species	8	16.858	2.107	4.558	0.001	54

### Influence of Seasonal Salt Stress on Morphological Properties

**Fig. 3:**
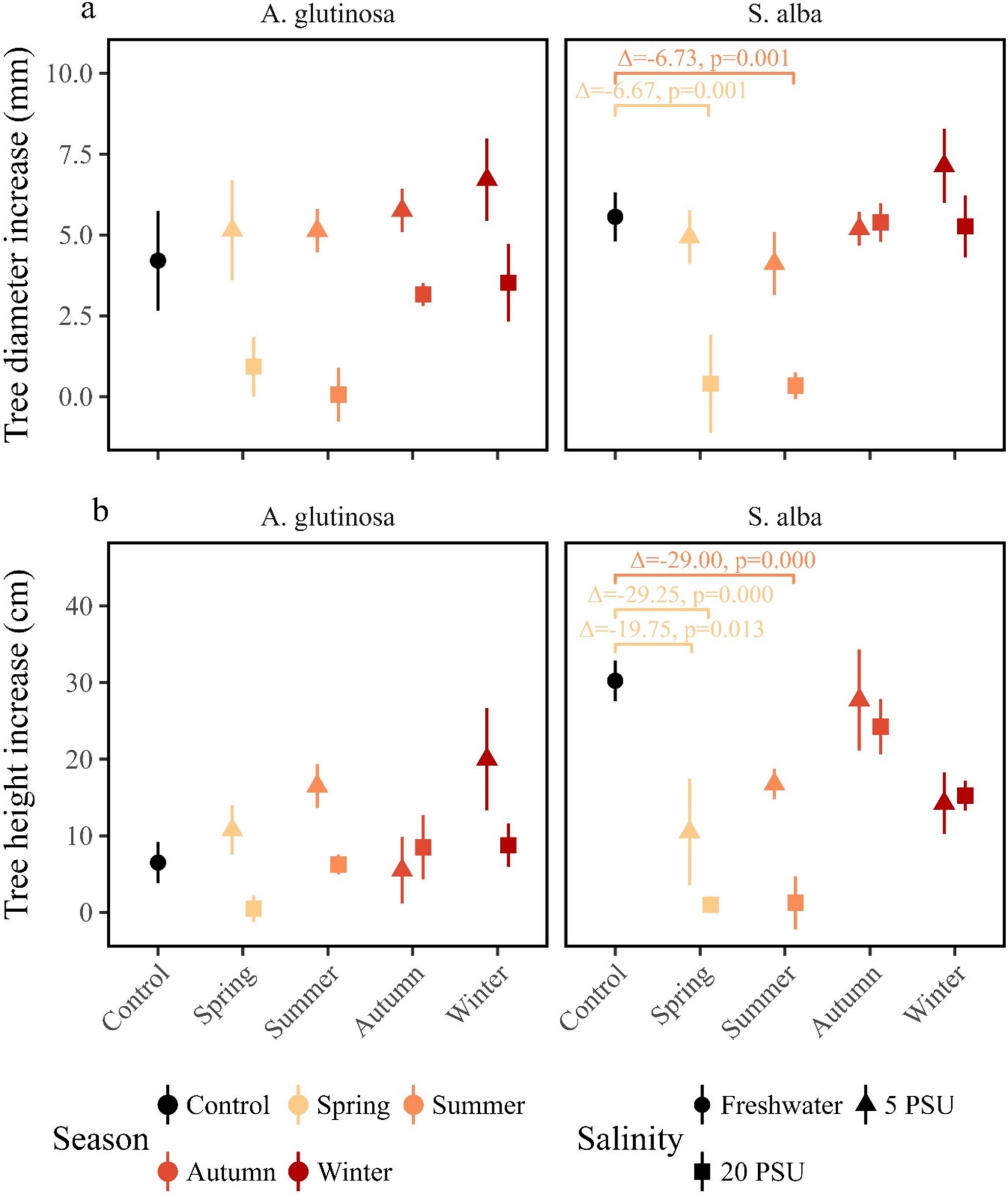
Changes in tree morphology (diameter and height) between the first measurement (April 2022) and the final measurement (May 2023) for the two species. (**a**) Diameter change: mean difference in trunk diameter (mean ± standard error). Colours encode the salinity treatment applied during the experiment (black = fresh‑water control; a gradient from yellow to red represents the sequence of treatments from spring to winter). Shape indicates the salinity intensity of the treatment (circle = fresh‑water control, triangle = 5 PSU, square = 20 PSU). The annotated values above the brackets are the mean differences (Δ) and the adjusted p‑values from the Dunnett post‑hoc test. An annotation is shown only for treatments with adjusted *p* < 0.05. (**b**) Height change: mean difference in tree height (mean ± standard error) plotted with the same colour and symbol coding as described for panel a

Growth responses to salinity differed markedly between *Alnus glutinosa* and *Salix alba* (Fig. 3; Tables 1 and 2). For stem diameter, *A. glutinosa* showed no meaningful response to any salinity treatment applied in spring, summer, autumn 2022, or winter 2023. Diameter increases under the 20 PSU treatment remained low and comparable to the control, with increases of 0.93 ± 0.93 mm in spring, 0.06 ± 0.83 mm in summer, and 4.21 ± 1.54 mm in the control (effect of treatment on diameter: F₈,₂₇ = 3.10, *p* = 0.013, *n* = 36; spring 20 PSU: mean difference = − 3.28, 95% CI [− 9.04, 3.28], *p* = 0.477; summer 20 PSU: mean difference = − 4.14, 95% CI [− 9.91, 4.15], *p* = 0.244, Table S2). In contrast, *S. alba* exhibited a strong reduction in diameter growth when exposed to 20 PSU salinity in spring and summer. Diameter growth in *S. alba* was only 0.40 ± 1.52 mm in spring and 0.34 ± 0.41 mm in summer, compared with 7.06 ± 1.60 mm in the control (effect of treatment on diameter: F₈,₂₇ = 6.38, *p* < 0.0001, *n* = 36; spring 20 PSU: mean difference = − 6.67, 95% CI [− 10.88, − 6.67], *p* = 0.001; summer 20 PSU: mean difference = − 6.73, 95% CI [− 10.94, − 6.73], *p* = 0.001).

**Table 2:** One-way ANOVA results on the last measures of the soil salinity in both soil layers, the last measures of Leaf Health Index, the tree growth in diameter and height (difference between the last and the first measure), the aboveground biomass, the upper and lower half of the belowground biomass

Measure	Term	DF	SS	MS	F	*P*	*N*. obs
Leaf Health Index	A. glutinosa	8	1.182	0.148	1.25	0.318	31
	S. alba	8	18.227	2.278	1542.275	< 0.0001	29
Soil salinity (mS), lower 12 cm	A. glutinosa	8	0.305	0.038	3.925	0.003	36
	S. alba	8	0.765	0.096	6.153	0	36
Soil salinity (mS), upper 12 cm	A. glutinosa	8	0.522	0.065	8.376	< 0.0001	36
	S. alba	8	0.592	0.074	5.806	0	36
Tree diameter	A. glutinosa	8	204.694	25.587	3.102	0.013	36
	S. alba	8	225.674	28.209	6.381	0	36
Tree height	A. glutinosa	8	1113.5	139.187	2.615	0.029	36
	S. alba	8	3541.389	442.674	6.661	< 0.0001	36
Dry aboveground biomass (g)	A. glutinosa	8	4.211	0.526	3.306	0.017	27
	S. alba	8	17.618	2.202	21.827	< 0.0001	27
Dry belowground biomass (g), lower 12 cm	A. glutinosa	8	2.178	0.272	0.881	0.55	27
	S. alba	7	13.51	1.93	9.773	0	23
Dry belowground biomass (g), upper 12 cm	A. glutinosa	8	6.227	0.778	1.904	0.122	27
	S. alba	8	57.329	7.166	13.897	< 0.0001	27

A similar contrast was observed for height growth. Height increase in *A. glutinosa* was not significantly affected by salinity, with no differences between the 20 PSU treatments and the control across seasons (effect of treatment on height: F₈,₂₇ = 2.62, *p* = 0.029, *n* = 36; spring 20 PSU: mean difference = − 6.00, 95% CI [− 20.63, -6.00], *p* = 0.787; summer 20 PSU: mean difference = − 0.25, 95% CI [− 14.88, 0.25], *p* = 1). By contrast, *S. alba* showed pronounced reductions in height growth under 20 PSU salinity in both spring and summer (effect of treatment on height: F₈,₂₇ = 6.66, *p* < 0.0001, *n* = 36). Height growth was reduced by 29.25 mm in the spring 20 PSU treatment and by 29.00 mm in the summer 20 PSU treatment relative to the control (spring: 95% CI [− 45.60, − 29.25], *p* < 0.0001; summer: 95% CI [− 45.35, − 29.00], *p* < 0.0001).

**Fig. 4:**
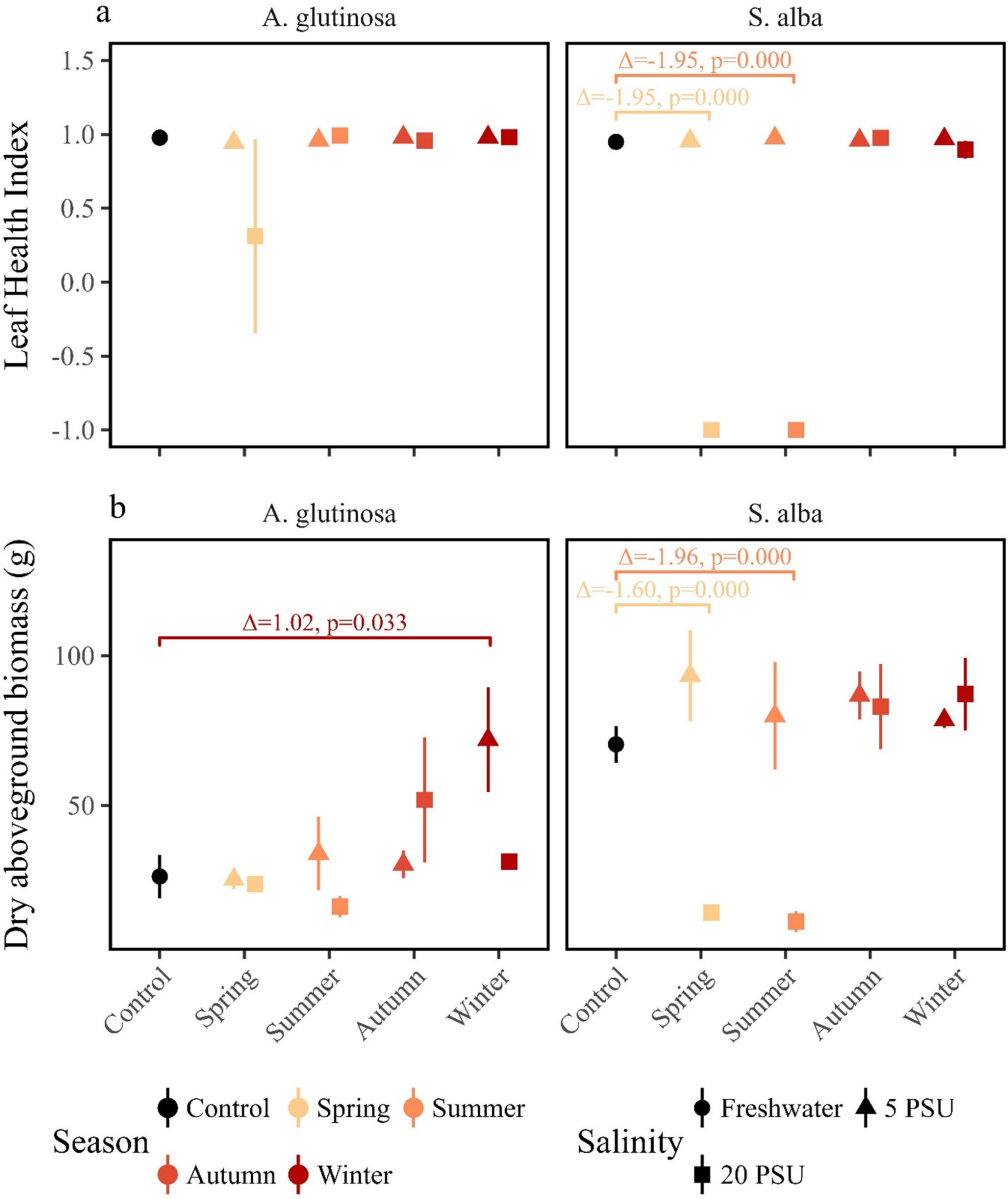
Mean values (± standard error) of the two response variables measured at the end of the experiment (15 May 2023) for the two tree species. (**a**) Leaf‑Health Index: average Leaf‑Health Index for each salinity regime. Colours indicate the salinity treatment applied during the experiment (black = fresh‑water control; a continuous gradient from yellow to red represents the sequence of salinity applications from spring through winter). Shape indicates the salinity intensity of the treatment (circle = fresh‑water control, triangle = 5 PSU, square = 20 PSU). The annotated values above the brackets are the mean differences (Δ) and the adjusted p‑values from the Dunnett post‑hoc test. An annotation is shown only for treatments with adjusted *p* < 0.05. (**b**) Above‑ground biomass: average above‑ground biomass (mean ± SE) for each salinity regime, plotted with the same colour and shape coding as described for panel a

The final Leaf Health Index values measured on 16 May 2023 further reflected these trends. *Alnus glutinosa* maintained values around 1 across all treatments, indicating that the treatments had no measurable effect (F_8,22_=1.25, *p* = 0.318, *n* = 31). In contrast, *S. alba* had index values of -1 in both spring and summer 20 PSU treatments, while all other treatments remained around 1 (F_8,20_=1542.28, *p* < 0.0001, *n* = 29, spring 20 PSU treatment: mean difference = -1.95, 95% CI [-1.95, -2.04], *p* < 0.0001; summer 20 PSU treatment: mean difference = -1.95, 95% CI [-1.95, -2.04], *p* < 0.0001; Fig. 4B, and Table 2 and S1 ), clearly indicating stress and reduced health. The observation of similar trends in all morphological properties within each species confirms the effects of the treatment and emphasises the differences in species’ responses to the timing and intensity of the salt-intrusion event.

### Influence of Seasonal Salt Stress on Biomass

*Alnus glutinosa* was not significantly affected by any salinity treatment, except for an observable increase in aboveground biomass in the winter 5 PSU treatment (*F*_8,18_ = 3.31, *p* = 0.017, mean difference = 1.02, 95% CI [1.02, 0.07], *p* = 0.033, Table 2 and S1). In contrast, *S. alba* showed a clear reduction in biomass in the spring and summer 20 PSU treatments (*F*_8,18_ = 12.83, *p* < 0.0001, spring 20 PSU treatment: mean ± SE = 14.28 ± 1.32 g, mean difference = -1.6, 95% CI [-1.60, -2.36], *p* < 0.0001; summer 20 PSU treatment: mean ± SE = 11.22 ± 3.51 g, mean difference = -1.96, 95% CI [-1.96, -2.72], *p* < 0.0001; control: mean ± SE = 70.43 ± 6.11 g; Fig. 4B, and Table 2 and S1). The belowground biomass follow a similar trend as the aboveground biomass, although less pronounced (effect of treatment on belowground biomass: *F*_8,36_ = 12.63, *p* < 0.0001 for the upper layer; *F*_8,33_ =5.05, *p* = 0 for the lower layer, Table 2, Figure S2a, and S2b). Additionally, the lower layer of the belowground biomass consistently had less biomass than the upper layer (e.g. in a 30–120 g range in the upper layer and 20–40 g range in the lower layer), and no root biomass was present in the lower layer of S. alba trees exposed to 20 PSU salinity in summer. Overall, the harvested above- and belowground biomass, particularly the measurable impact of the spring and summer 20 PSU treatments on *S. alba* and the insensitivity of *A. glutinosa*, highlights the differences between species in response to the timing and intensity of the salt-intrusion event, in line with the results from the non-destructively morphological characteristics.

## Discussion

In a future characterized by climate change, the fate of the remaining floodplain forests will be determined by salt intrusion events into estuaries, driven by storm surges or droughts (Herbert et al., 2015; White & Kaplan, 2017). Understanding how the timing of salt intrusion events affects the level of threat to floodplain forests vegetation is essential to ensure its conservation and future-proving management. The latter is especially important given that floodplain forests have been removed and altered by a long history of human exploitation, so that only few are remaining with a modified species composition (Havrdova et al., 2023). Here we show how the currently dominant *S. alba* is negatively affected by salt stress during the growing season (spring and summer), while the historically dominant *A. glutinosa* remains unaffected. Additionally, stress during the dormant season (autumn and winter) is insignificant for both species. This discrepancy may also be due to the plants’ lower evapotranspiration during their winter rest. This means that salinity can only enter the soil via diffusion rather than flowing to compensate for evaporation. This could potentially lead to reduced soil salinity, as measured in our results. This highlights the striking differences in salt sensitivity between these two dominant freshwater tree species and underscores that the sensitive species is only impacted by salt intrusion events during the growing season. Consequently, there is a risk of misestimating the threats of salt intrusion events if we do not account for both the seasonal sensitivity and species-specific sensitivity.

### Nature-Based Solutions Should Address Drought Impacts

Nature-based solutions (NBS) using wetlands and vegetated foreshores (green NBS) are gaining attention as sustainable alternatives to traditional grey infrastructure, particularly for addressing specific challenges such as flood and storm surge protection (Penning et al., 2023; Sahani et al., 2019; Siemes et al., 2023). However, many green NBS are designed with a narrow focus, often targeting a single or a few environmental issues, with drought impacts rarely considered during their development (Penning et al., 2023; Sahani et al., 2019; Yimer et al., 2024). To ensure these ecosystems continue to deliver their intended benefits, it is essential to understand and predict their long-term health and functionality (Bouma et al., 2014). Such predictions are becoming increasingly complex as climate change and direct human interventions introduce multiple, often simultaneous stressors. For instance, changing water regimes—characterized by higher water levels during storm surges and lower discharge during droughts—can lead to the dual stresses of altered river discharge and salt intrusion events (Costa et al., 2023; Lee et al., 2024). Our findings indicate that salt intrusion events during the growing season—when droughts and periods of low water availability are more common—can significantly harm sensitive freshwater species. In our study, plants were exposed to salinity for only one week, but natural droughts typically last much longer (Wegman et al., 2024). This is relevant since previous research has shown that the duration of salt exposure strongly influences its impact: brief intrusions are often less damaging, while prolonged exposure can result in severe and lasting effects on vegetation (Hootsmans & Wiegman, 1998; Li & Pennings, 2018; Saccon et al., 2025). However, previous research has shown that *A. glutinosa* is more tolerant of prolonged exposure to salinity than *S. alba* (Saccon et al., 2025). Research on other species and in different geographic regions has reported comparable seasonal influences (Vineis et al., 2011) and highlighted how interactions between salinity and hydrology affect species’ responses and recovery from salt stress (Poulter et al., 2008; Williams et al., 1999). Therefore, the ecological consequences of drought-driven salt intrusion are likely to be even more severe than those observed under our experimental conditions. Nature-based solutions and management strategies should thus focus on mitigating the effects of drought on salt intrusion, given its critical role in intensifying salt stress in freshwater ecosystems.

### Increasing Species Diversity as an Adaptive Strategy

In light of the growing challenges posed by climate change and human-induced stressors on ecosystem stability (Lemm et al., 2021; Palmer et al., 2009), current management practices that focus on maintaining single-species forest compositions may inadvertently increase vulnerability to salt intrusion events. This is particularly evident in the low-diversity floodplain forests, such as those currently present in much of western Europe, where forests are often dominated by one or two willow species propagated by cuttings. Current research indicates that these species are less salt-tolerant than the historically dominant *A. glutinosa*. Despite legal mandates for their conservation and restoration under the EU Council Directive 92/43/EEC (Article 17 web tool, (n.d.); Havrdova et al., 2023; Verhoeven, 2014), these habitats face increasing environmental threats. Our study, in line with previous studies on the same (Saccon et al., 2025) as well other plant species from other geographical areas (Conner et al., 1997; Hanley, et al., 2020a; Tolliver et al., 1997; White & Kaplan, 2017), highlights significant species-specific differences in salt tolerance, with critical implications for the conservation of floodplain forests. A shift toward climate resilient and adaptive strategies could provide a viable solution. Promoting species diversity and allowing for natural succession (Messier et al., 2019) offers a gradual transition from single-species freshwater forests to more resilient, mixed forests, provided that these include species tolerant to brackish conditions. This approach helps prevent widespread tree mortality and enhances biodiversity, enabling forests to better withstand new climate-induced stressors, possibly creating a long-term robust solution (Bouma et al., 2014; Isbell et al., 2015; Schuster et al., 2024). It ensures the continuous provision of essential ecosystem services, such as flood protection, nutrient removal, and carbon sequestration, without exposing bare soils to degradation (Berendse et al., 2015; Quijas et al., 2010). Adaptive strategies that use ecological diversity as a buffer against environmental change must be prioritised in future management efforts.

### The Role of Genetic Diversity in Stress Response

Historical reliance on vegetative propagation in European floodplain forests may have restricted genetic pools, compromising their long-term resilience. Research on this specific topic remains scarce, as highlited by Alimpić et al. (2022) in a survey of European conservation managers. This study revealed a systemic lack of genetic diversity assessments of riparian forests coupled with the prevailing perception that current species diversity is in a poor state. As with this study, previous research has not directly assessed the genetic variability of the target species, and there is still little empirical research on this topic. Existing data on *A. glutinosa* indicate high allelic diversity alongside low differentiation (Mingeot et al., 2016). Similarly, Sitzia et al. (2018) noted high genetic similarity and low differentiation in *S. alba*. These findings suggest extensive gene flow between populations, which is probably driven by water and wind dispersal. This creates corridors of genetically similar individuals along river systems (Beismann et al., 2000; Mosner et al., 2012; Rood et al., 2003). However, this connectivity has two implications: while high gene flow facilitates rapid recolonisation after stressful events (Sitzia et al., 2018), low genetic variability may reduce the resistance and survival of these forests when confronted with novel stressors (Fady et al., 2016). Failing to consider genetic factors when managing these forests could undermine biodiversity conservation efforts as well as the provision of ecosystem services (Alimpić et al., 2022). Therefore, in line with previous recommendations (Alimpić et al., 2022; Fady et al., 2016), future research should analyse how this reduced variation influences species’ responses to emerging stressors, and greater efforts should be directed toward transferring this knowledge to forest managers.

### Research Should Focus on Interactive Effects of Multiple Stressors

The interaction between salt intrusion and water level fluctuations, such as flooding during storm surges and low water levels during droughts, could further intensify environmental stressors affecting floodplain forest species. However, the combined impacts of drought, salt intrusion, and co-occurring stressors are still too often overlooked (Penning et al., 2023; Yimer et al., 2024). While our study did not evaluate these combined effects, previous research indicates that both *Alnus glutinosa* and *Salix alba* can tolerate repeated flooding (Glenz et al., 2006). However, *A. glutinosa* is relatively sensitive to drought, particularly during early growth stages before its roots can reach groundwater, due to its strong dependence on groundwater access (Claessens et al., 2010; Valor et al., 2020). In contrast, *S. alba*, adapted to disturbed environments, exhibits a greater tolerance to drought conditions (Karrenberg et al., 2002; Splunder et al., 1996). Although some studies have documented interactive effects of concurrent stressors on plant performance (for comprehensive overview see review by Parmesan & Hanley, 2015), empirical data are still scarce for the two riparian species examined here (*Alnus glutinosa* and *Salix alba*) under multiple, simultaneous stressors, creating uncertainty about their long-term resilience. The combined impact of salt intrusion and changes in water availability, such as those caused by drought, may be more severe than what is reflected in our findings. To better support conservation and restoration efforts, future research should prioritize understanding how these stressors interact, particularly in vulnerable ecosystems like floodplain forests.

## Conclusion

Understanding species-specific vegetation responses to seasonal dynamics of salt intrusion events is essential in order to protect the last remaining floodplain forests and to ensure the success of novel wetland restoration designs (for nature-based solutions or biodiversity goals). Our findings reveal that salt intrusion events occurring during summer (as typically caused by droughts) might pose a greater threat to wetland vegetation than those during winter (as typically caused by storm surge), with the impact being strongly species-specific. Increasing tree species diversity within floodplain forests is an important way forward to enhance ecosystem stability and resilience to environmental stresses. Diversifying these ecosystems will ensure their long-term effectiveness as natural flood defences and restore vulnerable wetland ecosystems in the face of climate change.

## Supplementary Information

Below is the link to the electronic supplementary material.

Supplementary Material 1 (DOCX 521 KB)

## Data Availability

The data of this study are openly available in the research repository 4TU: doi: 10.4121/c117b12a-e9e9-4279-84fa-1461219be451.
