# Supplementary material for: Floodplain Forests Are Sensitive to Salt-Intrusion During Summer Droughts When Dominated by Salix
Source: Estuaries Coast. 2026 May 7;49(4):113. doi: 10.1007/s12237-026-01726-1 (PMC13152930; doi:10.1007/s12237-026-01726-1)
Supplement: Supplementary file 1 — Supplementary Material 1 (DOCX 521 KB) [file 12237_2026_1726_MOESM1_ESM.docx]

07/01/2026

**Supplementary materials for:**

# Floodplain forests are sensitive to salt-intrusion during summer droughts when dominated by *Salix*

*Estuaries and Coasts*

Eleonora Saccon^1,2^*, Suzanne J.M.H. Hulscher^2^, Tjeerd J. Bouma^1,3^, Johan van de Koppel^1,4^

^1^ Royal Netherlands Institute for Sea Research (NIOZ), Department of Estuarine and Delta Systems, Yerseke, Netherlands

^2^ University of Twente, Department of Water Engineering and Management, Faculty of Engineering Technology, Enschede, Netherlands

^3^ Faculty of Geosciences, Department of Physical Geography, Utrecht University, 3508 TC Utrecht, The Netherlands

^4^ Conservation Ecology Group, Groningen Institute for Evolutionary Life Sciences, University of Groningen, 9700 CC Groningen, Netherlands

* Corresponding author: eleonora.saccon@nioz.nl

Table S1: two-way ANOVA on the initial measures of tree diameter, tree height and of Leaf Health Index.

| Measure | Term | DF | SS | MS | F | P | N. obs |
| --- | --- | --- | --- | --- | --- | --- | --- |
| Tree diameter | treatment | 7 | 13.75 | 1.964 | 0.216 | 0.98 | 64 |
|  | species | 1 | 39.063 | 39.063 | 4.305 | 0.043 | 64 |
|  | treatment:species | 7 | 65.437 | 9.348 | 1.03 | 0.423 | 64 |
| Leaf Health Index | treatment | 7 | 0.495 | 0.071 | 1.18 | 0.342 | 48 |
|  | species | 1 | 0.739 | 0.739 | 12.335 | 0.001 | 48 |
|  | treatment:species | 7 | 0.837 | 0.12 | 1.997 | 0.086 | 48 |
| Tree height | treatment | 7 | 854.484 | 122.069 | 1.114 | 0.37 | 64 |
|  | species | 1 | 28182.02 | 28182.02 | 257.211 | <0.0001 | 64 |
|  | treatment:species | 7 | 989.109 | 141.301 | 1.29 | 0.276 | 64 |


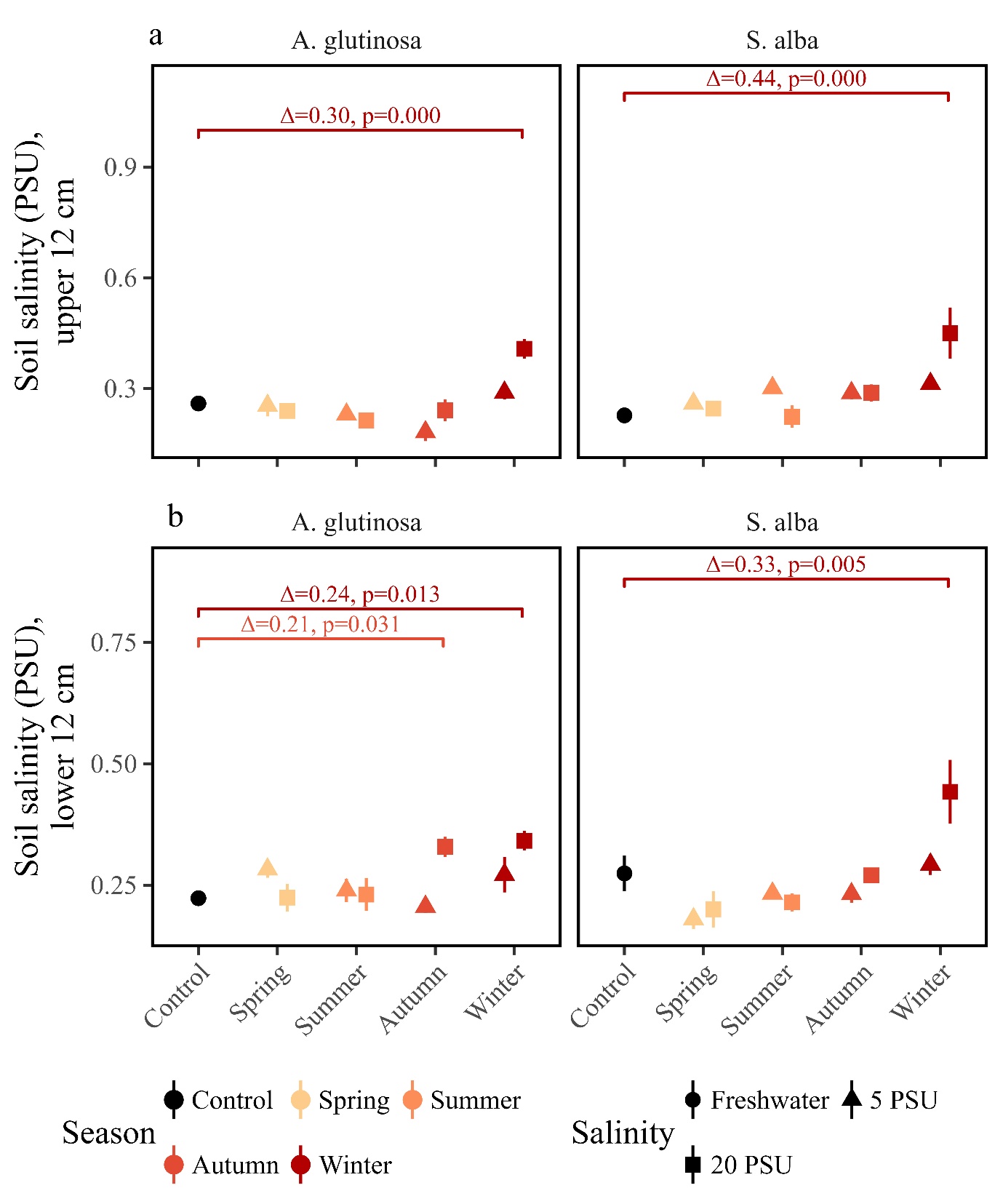
 **Fig. S1** Soil salinity (PSU) taken at the end of the experiment on 16/05/2023. a) Soil salinity in the upper half of the pots (mean ± standard error). Colours encode the salinity treatment applied during the experiment (black = fresh‑water control; a gradient from yellow to red represents the sequence of treatments from spring to winter). Shape indicates the salinity intensity of the treatment (circle = fresh‑water control, triangle = 5 PSU, square = 20 PSU). The annotated values above the brackets are the mean differences (Δ) and the adjusted p‑values from the Dunnett post‑hoc test. An annotation is shown only for treatments with adjusted p < 0.05. b) Soil salinity (PSU) in the lower half of the pots (mean ± standard error) plotted with the same colour and symbol coding as described for panel a.

**
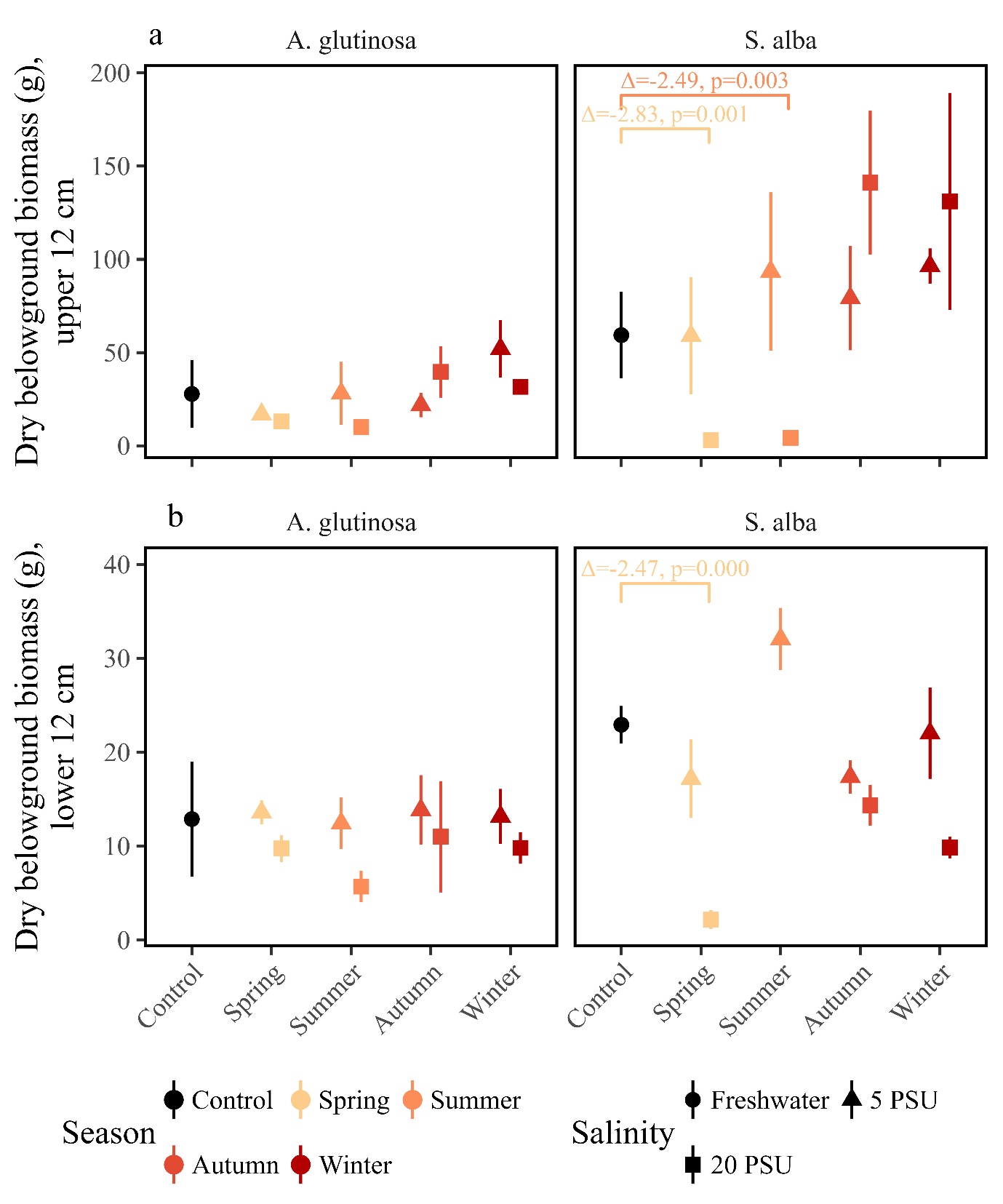
**

**Fig. S2** Dry belowground biomass. a) Belowground dry biomass from the upper half of the pots (mean ± SE). Colours indicate the salinity treatment applied during the experiment (black = fresh‑water control; a continuous gradient from yellow to red represents the sequence of salinity applications from spring through winter). Shape indicates the salinity intensity of the treatment (circle = fresh‑water control, triangle = 5 PSU, square = 20 PSU). The annotated values above the brackets are the mean differences (Δ) and the adjusted p‑values from the Dunnett post‑hoc test. An annotation is shown only for treatments with adjusted p < 0.05. b) Belowground dry biomass from the lower half of the pots (mean ± SE) for each salinity regime, plotted with the same colour and shape coding as described for panel a.

Table S2: Dunnet post -doc test between treatments for each species on the last measures of the soil salinity in both soil layers, the last measures of Leaf Health Index, the tree growth in diameter and height (difference between the last and the first measure), the aboveground biomass, the upper and lower half of the belowground biomass.

| Measure | Species | Contrast | Estimate | Adj.p.value | Conf.low | Conf.high |
| --- | --- | --- | --- | --- | --- | --- |
| Leaf  Health  Index | A. glutinosa | Spring 5 - Control 0 | -0.03 | 1 | -0.032 | -0.737 |
|  |  | Spring 20 - Control 0 | -0.67 | 0.106 | -0.666 | -1.427 |
|  |  | Summer 5 - Control 0 | -0.02 | 1 | -0.017 | -0.722 |
|  |  | Summer 20 - Control 0 | 0.01 | 1 | 0.014 | -0.747 |
|  |  | Autumn 5 - Control 0 | 0 | 1 | 0.004 | -0.757 |
|  |  | Autumn 20 - Control 0 | -0.02 | 1 | -0.02 | -0.725 |
|  |  | Winter 5 - Control 0 | 0 | 1 | 0.003 | -0.758 |
|  |  | Winter 20 - Control 0 | 0 | 1 | 0.003 | -0.758 |
|  | S. alba | Spring 5 - Control 0 | 0.01 | 1 | 0.007 | -0.084 |
|  |  | Spring 20 - Control 0 | -1.95 | <0.0001 | -1.949 | -2.04 |
|  |  | Summer 5 - Control 0 | 0.02 | 0.942 | 0.025 | -0.06 |
|  |  | Summer 20 - Control 0 | -1.95 | <0.0001 | -1.949 | -2.04 |
|  |  | Autumn 5 - Control 0 | 0.01 | 1 | 0.01 | -0.08 |
|  |  | Autumn 20 - Control 0 | 0.03 | 0.914 | 0.027 | -0.058 |
|  |  | Winter 5 - Control 0 | 0.02 | 0.988 | 0.019 | -0.071 |
|  |  | Winter 20 - Control 0 | -0.05 | 0.436 | -0.053 | -0.143 |
| Tree diameter | A. glutinosa | Spring 5 - Control 0 | 0.94 | 0.999 | 0.937 | -4.825 |
|  |  | Spring 20 - Control 0 | -3.28 | 0.477 | -3.277 | -9.04 |
|  |  | Summer 5 - Control 0 | 0.93 | 0.999 | 0.927 | -4.835 |
|  |  | Summer 20 - Control 0 | -4.14 | 0.244 | -4.145 | -9.908 |
|  |  | Autumn 5 - Control 0 | 1.55 | 0.967 | 1.552 | -4.21 |
|  |  | Autumn 20 - Control 0 | 1.53 | 0.97 | 1.53 | -4.233 |
|  |  | Winter 5 - Control 0 | 4.05 | 0.265 | 4.047 | -1.715 |
|  |  | Winter 20 - Control 0 | -0.68 | 1 | -0.682 | -6.445 |
|  | S. alba | Spring 5 - Control 0 | -2.13 | 0.602 | -2.127 | -6.343 |
|  |  | Spring 20 - Control 0 | -6.67 | 0.001 | -6.667 | -10.883 |
|  |  | Summer 5 - Control 0 | -2.95 | 0.269 | -2.95 | -7.166 |
|  |  | Summer 20 - Control 0 | -6.73 | 0.001 | -6.727 | -10.943 |
|  |  | Autumn 5 - Control 0 | -1.87 | 0.722 | -1.872 | -6.088 |
|  |  | Autumn 20 - Control 0 | -1.68 | 0.808 | -1.677 | -5.893 |
|  |  | Winter 5 - Control 0 | 1.11 | 0.971 | 1.11 | -3.106 |
|  |  | Winter 20 - Control 0 | -1.8 | 0.755 | -1.8 | -6.016 |
| Tree height | A. glutinosa | Spring 5 - Control 0 | 4.25 | 0.951 | 4.25 | -10.379 |
|  |  | Spring 20 - Control 0 | -6 | 0.787 | -6 | -20.629 |
|  |  | Summer 5 - Control 0 | 10 | 0.291 | 10 | -4.629 |
|  |  | Summer 20 - Control 0 | -0.25 | 1 | -0.25 | -14.879 |
|  |  | Autumn 5 - Control 0 | -1 | 1 | -1 | -15.629 |
|  |  | Autumn 20 - Control 0 | 2 | 1 | 2 | -12.629 |
|  |  | Winter 5 - Control 0 | 13.5 | 0.08 | 13.5 | -1.129 |
|  |  | Winter 20 - Control 0 | 2.25 | 0.999 | 2.25 | -12.379 |
|  | S. alba | Spring 5 - Control 0 | -19.75 | 0.013 | -19.75 | -36.101 |
|  |  | Spring 20 - Control 0 | -29.25 | 0 | -29.25 | -45.601 |
|  |  | Summer 5 - Control 0 | -13.5 | 0.14 | -13.5 | -29.851 |
|  |  | Summer 20 - Control 0 | -29 | 0 | -29 | -45.351 |
|  |  | Autumn 5 - Control 0 | -2.5 | 0.999 | -2.5 | -18.851 |
|  |  | Autumn 20 - Control 0 | -6 | 0.859 | -6 | -22.351 |
|  |  | Winter 5 - Control 0 | -16 | 0.057 | -16 | -32.351 |
|  |  | Winter 20 - Control 0 | -15 | 0.083 | -15 | -31.351 |
| Dry aboveground  biomass (g) | A. glutinosa | Spring 5 - Control 0 | 0.01 | 1 | 0.015 | -0.941 |
|  |  | Spring 20 - Control 0 | -0.05 | 1 | -0.046 | -1.002 |
|  |  | Summer 5 - Control 0 | 0.21 | 0.987 | 0.209 | -0.748 |
|  |  | Summer 20 - Control 0 | -0.46 | 0.624 | -0.458 | -1.414 |
|  |  | Autumn 5 - Control 0 | 0.19 | 0.992 | 0.192 | -0.765 |
|  |  | Autumn 20 - Control 0 | 0.6 | 0.353 | 0.6 | -0.356 |
|  |  | Winter 5 - Control 0 | 1.02 | 0.033 | 1.021 | 0.065 |
|  |  | Winter 20 - Control 0 | 0.24 | 0.973 | 0.237 | -0.719 |
|  | S. alba | Spring 5 - Control 0 | 0.26 | 0.877 | 0.26 | -0.502 |
|  |  | Spring 20 - Control 0 | -1.6 | <0.0001 | -1.596 | -2.358 |
|  |  | Summer 5 - Control 0 | 0.09 | 1 | 0.088 | -0.673 |
|  |  | Summer 20 - Control 0 | -1.96 | <0.0001 | -1.962 | -2.723 |
|  |  | Autumn 5 - Control 0 | 0.21 | 0.955 | 0.208 | -0.553 |
|  |  | Autumn 20 - Control 0 | 0.15 | 0.994 | 0.145 | -0.616 |
|  |  | Winter 5 - Control 0 | 0.12 | 0.999 | 0.116 | -0.646 |
|  |  | Winter 20 - Control 0 | 0.2 | 0.963 | 0.201 | -0.561 |
| Dry belowground  biomass (g),  lower 12 cm | A. glutinosa | Spring 5 - Control 0 | 0.27 | 0.992 | 0.266 | -1.067 |
|  |  | Spring 20 - Control 0 | -0.08 | 1 | -0.081 | -1.413 |
|  |  | Summer 5 - Control 0 | 0.13 | 1 | 0.133 | -1.2 |
|  |  | Summer 20 - Control 0 | -0.69 | 0.542 | -0.693 | -2.026 |
|  |  | Autumn 5 - Control 0 | 0.22 | 0.997 | 0.224 | -1.109 |
|  |  | Autumn 20 - Control 0 | -0.27 | 0.991 | -0.272 | -1.604 |
|  |  | Winter 5 - Control 0 | 0.18 | 0.999 | 0.18 | -1.153 |
|  |  | Winter 20 - Control 0 | -0.08 | 1 | -0.079 | -1.411 |
|  | S. alba | Spring 5 - Control 0 | -0.34 | 0.881 | -0.344 | -1.416 |
|  |  | Spring 20 - Control 0 | -2.47 | 0 | -2.47 | -3.669 |
|  |  | Summer 5 - Control 0 | 0.33 | 0.897 | 0.332 | -0.74 |
|  |  | Autumn 5 - Control 0 | -0.28 | 0.95 | -0.281 | -1.353 |
|  |  | Autumn 20 - Control 0 | -0.49 | 0.644 | -0.486 | -1.558 |
|  |  | Winter 5 - Control 0 | 0.45 | 0.705 | 0.453 | -0.619 |
|  |  | Winter 20 - Control 0 | -0.85 | 0.149 | -0.854 | -1.926 |
| Dry belowground  biomass (g),  upper 12 cm | A. glutinosa | Spring 5 - Control 0 | -0.09 | 1 | -0.085 | -1.618 |
|  |  | Spring 20 - Control 0 | -0.39 | 0.968 | -0.394 | -1.926 |
|  |  | Summer 5 - Control 0 | 0.08 | 1 | 0.079 | -1.454 |
|  |  | Summer 20 - Control 0 | -0.64 | 0.746 | -0.64 | -2.173 |
|  |  | Autumn 5 - Control 0 | 0.11 | 1 | 0.108 | -1.425 |
|  |  | Autumn 20 - Control 0 | 0.66 | 0.72 | 0.66 | -0.873 |
|  |  | Winter 5 - Control 0 | 0.96 | 0.356 | 0.959 | -0.574 |
|  |  | Winter 20 - Control 0 | 0.55 | 0.852 | 0.549 | -0.984 |
|  | S. alba | Spring 5 - Control 0 | 0.59 | 0.877 | 0.588 | -1.134 |
|  |  | Spring 20 - Control 0 | -2.83 | 0.001 | -2.825 | -4.547 |
|  |  | Summer 5 - Control 0 | 0.44 | 0.97 | 0.436 | -1.286 |
|  |  | Summer 20 - Control 0 | -2.49 | 0.003 | -2.493 | -4.215 |
|  |  | Autumn 5 - Control 0 | 0.92 | 0.517 | 0.917 | -0.805 |
|  |  | Autumn 20 - Control 0 | 1.35 | 0.166 | 1.351 | -0.371 |
|  |  | Winter 5 - Control 0 | 0.67 | 0.8 | 0.668 | -1.054 |
|  |  | Winter 20 - Control 0 | 1.13 | 0.313 | 1.126 | -0.596 |
